# Virulence-Associated Genes and Antimicrobial Resistance of *Aeromonas hydrophila* Isolates from Animal, Food, and Human Sources in Brazil

**DOI:** 10.1155/2020/1052607

**Published:** 2020-05-06

**Authors:** Emily Moraes Roges, Verônica Dias Gonçalves, Maira Duarte Cardoso, Marcia Lima Festivo, Salvatore Siciliano, Lucia Helena Berto, Virginia Leo de Almeida Pereira, Dalia dos Prazeres Rodrigues, Maria Helena Cosendey de Aquino

**Affiliations:** ^1^National Reference Laboratory Diagnosis of Enteric Bacteria, Oswaldo Cruz Institute, Oswaldo Cruz Foundation (FIOCRUZ), Brazil Avenue 4365, Rocha Lima Pavilion, 3rd floor, Room 316, Manguinhos, Rio de Janeiro, Rio de Janeiro 21040-360, Brazil; ^2^Post-graduate Program in Veterinary Hygiene and Animal Origin Food Processing, Department of Food Technology, Faculty of Veterinary Medicine, Federal Fluminense University (UFF), Vital Brasil Filho Street 64, Niterói, Rio de Janeiro 24230-340, Brazil; ^3^Post-graduate Program in Public Health and Environment, Sérgio Arouca National School of Public Health (ENSP), Oswaldo Cruz Foundation, Brazil Avenue 4365, Manguinhos, Rio de Janeiro, Rio de Janeiro 21040-360, Brazil; ^4^General Coordination of Public Health Laboratories (CGLAB), SRTVN–FRAME 701, Block “D”, 6th floor, Brasilia/Federal District 70719-040, Brazil

## Abstract

Aeromonads are natural inhabitants of aquatic environments and may be associated with various human or animal diseases. Its pathogenicity is complex and multifactorial and is associated with many virulence factors. In this study, 110 selected *Aeromonas hydrophila* isolates isolated from food, animals, and human clinical material from 2010 to 2015 were analyzed. Antimicrobial susceptibility testing was performed by the disk diffusion method, and polymerase chain reaction was conducted to investigate the virulence genes hemolysin (*hly*A), cytotoxic enterotoxin (*act*), heat-labile cytotonic enterotoxin (*alt*), aerolysin (*aer*A), and DNase-nuclease (*exu*). At least 92.7% of the isolates had one of the investigated virulence genes. Twenty different virulence profiles among the isolates were recognized, and the five investigated virulence genes were observed in four isolates. Human source isolates showed greater diversity than food and animal sources. Antimicrobial resistance was observed in 46.4% of the isolates, and multidrug resistance was detected in 3.6% of the isolates. Among the 120 isolates, 45% were resistant to cefoxitin; 23.5% to nalidixic acid; 16.6% to tetracycline; 13.7% to cefotaxime and imipenem; 11.8% to ceftazidime; 5.9% to amikacin, gentamicin, and sulfamethoxazole-trimethoprim; and 3.9% to ciprofloxacin and nitrofurantoin. Overall, the findings of our study indicated the presence of virulence genes and that antimicrobial resistance in *A*. *hydrophila* isolates in this study is compatible with potentially pathogenic bacteria. This information will allow us to recognize the potential risk through circulating isolates in animal health and public health and the spread through the food chain offering subsidies for appropriate sanitary actions.

## 1. Introduction

Since its first isolation in 1890, several events have discussed numerous aspects of the genus *Aeromonas*, and some of these, which have taken place over the past century, have been instrumental in understanding current issues about this group of bacteria [1].

*Aeromonas* microorganisms are highly adaptable to aquatic environments and have been described as pathogenic to humans and animals. The genus *Aeromonas* comprises more than 30 valid species, of which *A*. *hydrophila*, *A*. *caviae*, *A*. *media*, *A*. *veronii* bv*. sobria*, and *A*. *veronii* bv*. veronii* are of particular clinical significance [2].

They are widely isolated from clinical, environmental, and food samples where they can develop even at low temperatures and produce toxins, which significantly increase the risk of foodborne infection [3, 4].

Aeromonads have a wide geographical distribution, being able to determine infections in animals and humans [5]. Commonly found in aquatic environments, they are recognized as eventual pathogens of reptiles, fish, and some mammalian species. Recognized as emerging pathogens, their situation is privileged when natural disasters occur, having been largely isolated from skin and soft tissue infections in tsunami survivors that struck Thailand in 2004 [6]. Besides, *Aeromonas* have been recognized as a relevant etiological agent in human gastrointestinal infections, having been isolated from food and drinking water samples [7, 8].

Its virulence is multifactorial, and numerous factors have been identified in intestinal and systemic infections caused by this microorganism including endotoxins, enterotoxins, adhesins, cytotoxins, hemolysins, lipases, and proteases [7, 9].

*Aeromonas* spp. have the ability to receive and transmit a set of genes located within genetic elements such as plasmids, IS elements, transposons, genomic and/or pathogen islands, and integron-associated gene cassettes. These, referred to as flexible, can encode virulence factors, toxic compounds, and antibiotic resistance [10]. These elements are important in the rapid transfer of genetic materials into the microbial community. Environmental contamination is considered the most efficient for the selection of resistant populations as well as for the exchange of resistant genes through mobile genetic elements [11].

In recent years, the relevance of continuous isolation and identification of *A*. *hydrophila* observed in National Reference Laboratory for Bacterial Enteroinfections from Oswaldo Cruz Institute, especially in migratory marine mammal species that land on the Brazilian coast, has been questioned. The possibility of introducing different genetic traits through transfer to ubiquitous species in our environment is the fundamental concern. In contrast, the lack of literature in our country regarding the relevance of such microorganisms impels the need for subtyping and identification of virulence and antimicrobial drug resistance characteristics.

Based on the current available knowledge about this microorganism, this study is aimed at investigating a group of virulence-associated genes and antimicrobial resistance profiles in *Aeromonas hydrophila* isolated from animal, food, and human sources in order to characterize circulating isolates in Brazil and contribute to the knowledge of its relevance to animal and public health.

## 2. Methods

### 2.1. Selection of *Aeromonas hydrophila* Isolated in NRLED

110 *Aeromonas hydrophila* isolates from food (*n* = 28) (meat, fish, and chicken), animal (*n* = 52) (seabirds, marine mammals, and chelonians), and human clinical sources (*n* = 30) (Table 1) from 2010 to 2015 were analyzed at National Reference Laboratory for Enteric Diseases (NRLED), Oswaldo Cruz Institute, FIOCRUZ. The food samples were from ready-to-eat plate (meat and chicken) linked to foodborne disease. Fishes and scallops were from their natural habitat, and the marine animal isolates were obtained through monitoring programs carried out at FIOCRUZ. The human samples were obtained from patients with clinical symptoms and sent to NRLED by Public Health Laboratories.

### 2.2. Biochemical Characterization

Isolation and identification were performed according to Janda and Abbott [12]. The isolates were sown in Glutamate Starch Phenol-Red Agar medium (Merck) and screened in Kligler Iron Agar (Difco) and Lysine Iron Agar (Difco) and identified to the specie level by nonautomatized biochemical tests. They presented a positive oxidase test, and Vibriostatic Agent O/129 test showed resistance in 10 *μ*g and 150 *μ*g concentrations, as shown by Martin-Carnahan and Joseph [13].

### 2.3. Genus *Aeromonas* Identification Using the GCAT-PCR (237 bp)

The Glycerophospholipid-Cholesterol Acyltransferase (*gcat*) gene was amplified using a primer pair as reported previously [14]. The presence of this gene (237 bp) was visualized on 2% agarose gel (Sigma) stained with ethidium bromide.

### 2.4. Determination of Antimicrobial Susceptibility

Antimicrobial susceptibility testing was performed by the disk diffusion method according the Clinical and Laboratory Standard Institute (CLSI) recommendations for *Aeromonas* species (CLSI M45, 3^rd^ ed., 2015) [15] and for *Enterobacteriaceae* (CLSI M100, 29^th^ ed., 2019) [16] to the antimicrobials nalidixic acid (NAL) 30 *μ*g, amikacin (AMK) 30 *μ*g, ceftazidime (CAZ) 30 *μ*g, cefoxitin (FOX) 30 *μ*g, ceftriaxone (CTX) 30 *μ*g; ciprofloxacin (CIP) 5 *μ*g, chloramphenicol (CHL) 30 *μ*g; gentamicin (GEN) 30 *μ*g, imipenem (IPM) 10 *μ*g, nitrofurantoin (NIT) 300 *μ*g, sulfamethoxazole-trimethoprim (SXT) 1.25/23.75 *μ*g, and tetracycline (TCY) 30 *μ*g. *Escherichia coli* ATCC 25922 was used for quality control of the antimicrobial susceptibility test.

### 2.5. Polymerase Chain Reaction (PCR) of Virulence Genes

DNA extraction was performed using commercial kit (DNA DNeasy Tissue, Qiagen) following the manufacturer instructions. DNA amplification step was conducted, in order to investigate the virulence genes hemolysin (*hly*A–597 bp) [17], cytotoxic enterotoxin (*act*–232 bp), heat-labile cytotonic enterotoxin (*alt*–442 bp) [18], aerolysin (*aer*A–431 bp), and DNase-nuclease (*exu*–323 bp) [19]. Eight microliters of PCR product mixed with 5x gel loading dye was loaded onto an agar gel 2% (Sigma) in 0.5x Tris-Borate-EDTA buffer, and a 100 bp DNA ladder (Invitrogen by Thermo Fischer Scientific) was used as a molecular weight marker. Gels were visualized by a UV transilluminator (ImageQuant).

## 3. Results

### 3.1. Biochemical and Molecular Confirmation of *Aeromonas hydrophila* Isolates

The isolates have been confirmed for the *Aeromonas* genus by detection of the *gcat* gene present in all 110 isolates and for the *A. hydrophila* species through the use of nonautomated biochemical tests, whose results were compatible with the investigated species.

### 3.2. Distribution of *Aeromonas hydrophila* according to the Sources

Most isolates were from marine animal source, and migratory mammals showed the highest isolation percentage (81.1%). Among the food samples, isolates from fish represented 67.8%. Human *A*. *hydrophila* isolates from gastroenteric infections corresponded to 86.7%, and the sources of infections were unknown. The distribution is shown in Table 1.

### 3.3. Distribution of Virulence Genes

**Figure 1:**
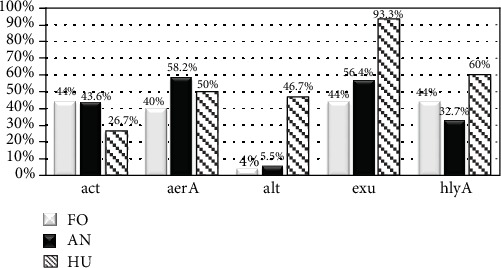
Percentage distribution of virulence genes in *Aeromonas hydrophila* according to isolation source. ^∗^FO: food; AN: animal; HU: Human. ^∗∗^*act*: cytotoxic enterotoxin; *aer*A: aerolysin; *alt*: heat-labile cytotonic enterotoxin; *exu*: DNase-nuclease; *hly*A: hemolysin.

Among the studied isolates, 92.7% (102) presented at least one of the virulence genes distributed among the 20 virulence profiles, highlighting 17 isolates that simultaneously presented 4 to 5 virulence genes (see Table 2). Considering the isolates of human origin among 30 *A*. *hydrophila*, we found 11 different virulence profiles with 1 to 5 virulence genes (see Table 3). The *act*, *aer*A, *alt*, *exu*, and *hly*A genes were detected in 36, 57, 18, 70 and 47 *A*. *hydrophila* isolates, respectively. The frequencies of all the gene encoding virulence factors according to the source of studied Aeromonads are shown in Figure 1.

### 3.4. Antimicrobial Susceptibility

Almost 53.6% of the isolates were susceptible to all tested antibiotic. The remaining isolates showed resistance to at least one antimicrobial drug. Resistance to one or two antibiotics was observed in 40% of resistant isolates; however, 6.4% of resistant isolates were resistant to three or more antibiotics. Overall, twenty-one different resistance profiles were identified. Among these 51 resistant isolates, the distribution of antimicrobial resistance rates can be observed Table 4.

## 4. Discussion

In this present study, it was possible to observe the diversity of virulence markers presented by the isolates, through the virulence profiles. Twenty virulence profiles were observed, and each profile had one to five genes. Virulence factors of a bacterium are often associated with the damage it causes to the host. Certain studies suggest that some *Aeromonas* species synthesize more virulence factors and more frequently, showing clonal origin of virulence; so, only a few clones would be responsible for disease progression [20–22].

The diversity in virulence profiles of isolates and the relationship between virulence markers show that they vary according to the needs for their survival in an environment. Rasmussen-Iveyi et al. [23] mention that the expression of virulence factors is linked to gene regulation cascades associated with interactions of microorganisms with the environment in which they are found.

*Aeromonas* pathogenicity is complex and multifactorial and is associated with many virulence factors, and there is not a definitive link between the presence of specific toxin genes and clinical presentation [24].

In this study, the selection of isolates from different sources in the food chain allowed the observation of different virulence factor combinations, confirming the multifactorial virulence profile in *Aeromonas* spp. Among the tested virulence genes, *hly*A, *aer*A, and *exu* were the most common genes and the *exu* gene was the most prevalent, present in 63.7% of the isolates. The genes *aer*A, *exu*, and *hly*A showed a higher percentage of positivity among the isolates from human source, and *exu* was the most prevalent. Each of these genes plays an important role in the pathogenicity observed in diarrheal diseases [1, 25]. Despite *Aeromonas* harboring different numbers and types of virulence genes, in this study, among the five investigated genes, a correlation between clinical symptoms and gene encoding virulence factors was not observed However, the *exu* gene was present at 93.3% of intestinal and extraintestinal infections. The *exu* gene codes for an extracellular DNase which blocks the antibacterial host defenses [26]. Its presence is associated with the microbial capacity of invasion and colonization, as well as evasion of the host immune system [9]. The high percentage of positive isolates for this gene enhances its relevance for the maintenance of *Aeromonas* spp. in the host.

Clinical isolates harboring a variety of toxin genes have been reported [3, 23]. Patients with different clinical manifestations brought more attention to some virulence factors, including hemolysins, enterotoxins, cytotoxic enterotoxin (act), and extracellular enzymes (proteases, amylases, lipases, ADP-ribosyltransferases, and DNases) [21].

In intestinal infections, *alt* has been reported to be associated with loose stool, *alt* plus *ast* with watery stools, and *act* with bloody diarrhea [22]. The heat-labile cytotonic enterotoxin *alt* is responsible for causing significant fluid secretion in the host's cell [23]. In this study, although ten diarrheic stools samples were associated with the presence of *alt*, 15 samples of diarrheic stools were not related with *alt*. In addition, a relationship between infection and presence of gene encoding virulence factors was not observed and might be related to the limited number of isolates from extraintestinal infections. Likewise, a study completed by Wu et al. [27] found no direct association between the presence of the genes *aer*A, *hly*A, *alt*, and *ast*, in *Aeromonas* isolates and development of extraintestinal infections or bacteremia.

Castelo-Branco et al. [28] observed that Aeromonads of clinical origin had fewer virulence genes than those isolated from other sources. In our study, it was possible to observe variations in the relationship between virulence genes and source, thus indicating that the distribution of virulence genes among *Aeromonas* is not uniform.

Except for the *alt* gene, observed in only one isolate (AhF1 from chicken), *act*, *exu*, and *hly*A genes were found with the same frequency (44%) and *aer*A in 40% in the foodborne isolates. Raw seafood corresponded to 82% of the samples analyzed and had all *hly*A-positive samples among isolates of food origin.

Among the food and animal samples, the same percentage of *act* gene was observed, which is 44% and 43.6%, respectively. Rather et al. [29] found 82% positivity for the *act* gene among isolates from different water sources and fish. Cytotonic enterotoxin encoded by this gene is responsible for triggering inflammatory response in host cells, plasma membrane disorders, and intestinal villus degeneration in cases of bloody diarrhea [1].

Among the animal isolates, *aer*A and *exu* were the most frequently observed genes. The *exu* gene was observed in 72.5% of marine animals with migratory characteristics. Among the 10 seabirds evaluated, this gene was only present in one *Leucopheus atricilla* isolate. Overall, the results showed high prevalence of the gene *exu* among the isolates evaluated at this study. Khor et al. [30] highlight that 96% of *Aeromonas* sp. environmental isolates presented the *exu* gene, corroborating the prevalence and emphasizing the importance of this gene for the survival of the bacteria.

Some animal specimens (*n* = 15) included in this study were from beached whales. All the 15 *Aeromonas hydrophila* isolates from these animals were positive for the *exu* gene, and 10 of them were positive for the *aer*A gene. Aerolysin (*aer*A) is the major contributor to the virulence of pathogenic *Aeromonas* isolates [31]. Aerolysin is a pore-forming toxin that binds to receptors on the target cell membrane. After proteolytic activation, this toxin induces pore or channel formation, leading to the destruction of membrane permeability, osmotic lysis, and cell death. [32]. The evaluation of the gene *aer*A was the most prevalent gene in the studied marine mammals (73.8%); from a human source, it was 50% and from a food source, it was 35.7%, which was identical to the findings of previous reports [33, 34].

Pereira et al. [35] found a frequency of approximately 20% in the isolation of *Aeromonas* in marine mammals from the south and southeast coast of Brazil. The presence of these microorganisms in aquatic migratory animals and the fact that presenting virulence factors can also found in isolates of human origin show zoonotic characteristics in *Aeromonas* spp. Several *Aeromonas* spp. have been reported as important zoonotic pathogens based on their virulence and antibiotic resistance profiles [12].

Data from the Brazilian Ministry of Health [36] have shown that animal-derived food, such as fish, triggers outbreaks of food- and water-borne diseases (FWBD). Water is the natural habitat of these bacteria and an important source of food contamination. *Aeromonas* spp. in food are a predominant feature in fish consumption, even though it has been studied in swine, chickens, and humans. It may occur in the excrement of infected animals and sick people who handle food.

Given the risk to human health, the incidence of antimicrobial resistance is alarming, particularly among *A*. *hydrophila*, *A*. *caviae*, and *A*. *sobria,* which are considered pathogens responsible for infections in both fish and humans [37]. These bacteria may be resistant by carrying intrinsic genes or by acquiring resistance markers from other microorganisms [4, 8]. Studies demonstrate that *Aeromonas* spp. can acquire resistance during treatment, as presented with tetracycline used in the treatment of bacterial infections in fish [38]. Increased resistance to antibiotics in *Aeromonas* species from different sources has been reported, showing appearance not only in isolates of clinical origin but also from other sources of isolation such as fish, food, and natural waters [4, 8].

In this study, 33.3% of samples from human source, 39.3% from food, and 63.5% from animal source were susceptible to all antimicrobials. All isolates isolated from marine animals from 2010 to 2013 were susceptible to all antimicrobial drugs, a condition that changed from 2014 on marine mammals and seabirds. Among 21 samples (2014 to 2015), 19 were resistant to at least one antimicrobial drug.

Multidrug resistance was observed in 16.7% of isolates from the human source, including fluoroquinolone and carbapenens. In food origin samples, the resistance profile was present in isolates since 2010, and one fish isolate was multiresistant (CAZ, CTX, IPM, NAL, and TCY). It has also been perceived that the fish farms analyzed either lacked proper water management or there was no management at all, with the consequent water and fish contamination. Lack of water management may cause disease and even death in fish. Because of faulty knowledge and inadequate manpower, producers use antimicrobials indiscriminately, causing the proliferation of antibiotic-resistant or even multidrug-resistant bacteria to antimicrobials [39]. According to Souza et al. [40], there is very scanty information on fish farming management and its consequences on water quality and on the health of fish in fish farms.

*Aeromonas* spp. are usually isolated from patients suffering from traveller's diarrhea. Hofer et al. [41] detected the pathogen as the cause of traveller's diarrhea in 18 (2%) out of 863 patients. A study on the outbreak of diarrhea in the town of São Bento do Una PE, Brazil, revealed that 114 (19.5%) out of the 582 coprocultures performed among the 2170 registered cases were caused by *Aeromonas* spp. [41].

Aeromonads were initially described as susceptible to tetracycline, chloramphenicol, cephalosporins, aminoglycosides, and quinolones [4, 12]. However, chromosomal inducible *β*-lactamases are recognized as a major mechanism of resistance to antimicrobials in *Aeromonas* spp. These enzymes are widely distributed among *Aeromonas* microorganisms, those of class C, as they confer resistance to cephalosporins and cefoxitin [4, 42]. Considering this, the cefoxitin resistance found in this study could be justified by the probable action of the enzyme.

Quinolone resistance has also been reported in *Aeromonas*. Among the studied isolates, 12 were resistant to nalidixic acid and 2 to ciprofloxacin. Sinha [43] reported high levels of intrinsic resistance to antimicrobial drugs. Resistance to these drugs may be related to *gyr* genes of chromosomal origin and *qnr* of plasmid origin. Previous studies [44–46] have identified *Aeromonas* spp. showing resistance to quinolones in domestic and free-living animals, hospital effluents, and wastewater. These results suggest the role of Aeromonads in the dissemination of antimicrobial resistance.

The presence of cephalosporin-, quinolone-, and carbapenem-resistant isolates are among the isolate points to the search for genes that characterize antimicrobial resistance. Acquiring and spreading antibiotic resistance genes (ARG) are of particular significance, as it is important for the health of humans and animals [10]. Aeromonads may become a reservoir of gene encoding resistance to antimicrobial drugs; studies showed the spread of tetracycline-resistant plasmids between *A. hydrophila* and *E. coli* as well as between human and aquaculture in different geographical regions [47]. The study of the resistome in different levels, such as phenotype, genotype, genomic, and epidemiological level, has turned into an important approach to understand the origin of the antibiotic resistance and its relationship with horizontal gene transfer in the genus *Aeromonas* spp., which is a pathogen related to public health problems [4].

## 5. Conclusion

In conclusion, the observation of varying virulence profiles shows the ability of *Aeromonas hydrophila* to adapt to the conditions of its environment. The *Aeromonas* isolates in this study present virulence and antimicrobial resistance aspects compatible with potentially pathogenic species capable of transferring the genes responsible for antibiotic resistance to other pathogenic microorganisms in humans and throughout the food chain which is a risk to human and animal health.

## Figures and Tables

**Table 1:** *Aeromonas hydrophila* distributed among different isolation sources and Brazilian geographic area.

Source		No.	Geographic area^∗^
Human (*n* = 30)	Blood	1	ST
	Fecal swab	17	NE (2), ST (1), SE (14)
	Feces	9	NE (3), ST (5), SE (1)
	Lung	1	MW
	Secretion	1	ST
	Synovial fluid	1	MW

Food (*n* = 28)	Meat (*Bos taurus*)	1	SE
	Chicken (*Gallus gallus*)	4	SE
	Scallop (*Pecten maximus*)	4	SE
	Fish (*Genidens barbus*)	2	SE
	Fish (*Rachycentron canadum*)	13	SE
	Fish (*Mugil liza*)	3	SE
	Fish (*Oreochromis niloticus*)	1	SE

Animal^A^ (*n* = 52)	*Arctocephalus gazella* ^1^	4	SE
	*Ardea cocoi* ^2^	1	SE
	*Chelonia mydas* ^3^	1	SE
	*Eretmochelys imbricata* ^4^	1	SE
	*Eubalaena australis* ^5^	5	SE
	*Leucophaeus atricilla* ^6^	5	SE
	*Lontra longicaudis* ^7^	1	SE
	*Megaptera novaeangliae* ^8^	10	SE
	*Pontoporia blainvillei* ^9^	3	ST (1), SE (2)
	*Stenella coeruleoalba* ^10^	7	ST
	*Sterna hirundinacea* ^11^	2	SE
	*Sula leucogaster* ^12^	2	SE
	*Trichechus manatus* ^13^	10	SE

^∗^Brazilian geographic areas: MW: midwest; NE: northeast; ST: south; SE–southeast. ^A^The popular names. ^1^Artic fur seal. ^2^Cocoi heron. ^3^Green sea turtle. ^4^Hawksbill sea turtle. ^5^Southern right whale. ^6^Laughing gull. ^7^Neotropical otter. ^8^Humpback whale. ^9^La Plata dolphin. ^10^Striped dolphin. ^11^South American tern. ^12^Brown booby. ^13^West Indian manatee.

**Table 2:** Number of *Aeromonas hydrophila* isolates with 4 to 5 virulence genes by source and origin.

Virulence profile	No. isolates	Source	Origin (*n*)
*act*, *aer*A, *alt*, *exu*, *hly*A	4	AN HU	*A*. *gazella* (1) Fecal swab (1) Feces (2)
*act*, *aer*A, *alt*, *exu*	1	FO	*Gallus gallus*
*act*, *aer*A, *alt*, *hly*A	1	AN	*M. novaeangliae*
*act*, *aer*A, *exu*, *hly*A	11	AN HU	*P*. *blainvillei* (2) *S*. *coeruleoalba* (7) *L*. *atricilla* (1) Fecal swab (1)

^∗^FO: food; AN: animal; HU: human. ^∗∗^*act*: cytotoxic enterotoxin; *aer*A: aerolysin; *alt*: heat-labile cytotonic enterotoxin; *exu*: DNase-nuclease; *hly*A: hemolysin.

**Table 3:** Virulence profiles of *Aeromonas hydrophila* isolated from human sources.

Isolate	Source	Virulence profile
AhH1	Blood	*Exu*
AhH2	Diarrheic stools	*Act*, *aerA*, *Exu*
AhH3	Diarrheic stools	*aerA*, *Exu*, *hlyA*
AhH4	Diarrheic stools	*aerA*, *Exu*, *hlyA*
AhH5	Diarrheic stools	*aerA*, *Exu*, *hlyA*
AhH6	Diarrheic stools	*Exu*
AhH7	Diarrheic stools	*aerA*, *Exu*
AhH8	Diarrheic stools	*hlyA*
AhH9	Diarrheic stools	*hlyA*
AhH10	Diarrheic stools	*Act*, *aerA*, *alt*, *Exu*, *hlyA*
AhH11	Diarrheic stools	*Act*, *aerA*, *Exu*, *hlyA*
AhH12	Diarrheic stools	*aerA*, *alt*, *Exu*, *hlyA*
AhH13	Diarrheic stools	*Alt*, *Exu*, *hlyA*
AhH14	Diarrheic stools	*Act*, *aerA*, *Exu*
AhH15	Diarrheic stools	*Act*, *alt*, *Exu*
AhH16	Diarrheic stools	*Alt*, *Exu*, *hlyA*
AhH17	Diarrheic stools	*Alt*, *Exu*, *hlyA*
AhH18	Diarrheic stools	*Exu*
AhH19	Diarrheic stools	*aerA*, *Exu*, *hlyA*
AhH20	Diarrheic stools	*aerA*, *Exu*, *hlyA*
AhH21	Diarrheic stools	*aerA*, *Exu*, *hlyA*
AhH22	Diarrheic stools	*Alt*, *Exu*
AhH23	Diarrheic stools	*Alt*, *Exu*
AhH24	Diarrheic stools	*Alt*, *Exu*
AhH25	Diarrheic stools	*Act*, *aerA*, *alt*, *Exu*, *hlyA*
AhH26	Diarrheic stools	*Act*, *aerA*, *alt*, *Exu*, *hlyA*
AhH27	Diarrheic stools	*Alt*, *Exu*
AhH28	Lung	*Alt*, *Exu*, *hlyA*
AhH29	Secretion	*Exu*
AhH30	Synovial fluid	*aerA*, *alt*, *Exu*, *hlyA*

^∗^ *act*: cytotoxic enterotoxin; *aer*A: aerolysin; *alt*: heat-labile cytotonic enterotoxin; *exu*: DNase-nuclease; *hly*A: hemolysin.

**Table 4:** Antibiotic resistance patterns in *Aeromonas hydrophila* distributed by source.

Antimicrobial drug	Total (*n* = 110)		Human (*n* = 30)		Food (*n* = 28)		Animal (*n* = 52)	
	*N*	%	*N*	%	*N*	%	*N*	%
Amikacin (AMK)	3	2.7	2	6.7	0	0	1	1.9
Cefoxitin (FOX)	29	26.4	5	16.7	11	39.3	13	25.0
Ceftazidime (CAZ)	9	8.2	2	6.7	7	25.0	0	0
Ceftriaxone (CTX)	7	6.4	6	20.0	1	3.6	0	0
Chloramphenicol (CHL)	0	0	0	0	0	0	0	0
Ciprofloxacin (CIP)	2	1.8	2	6.7	0	0	0	0
Gentamicin (GEN)	3	2.7	3	10.0	0	0	0	0
Imipenem (IPM)	7	6.4	2	6.7	3	10.7	2	3.8
Nalidixic acid (NAL)	14	12.7	7	23.3	4	14.3	3	5.8
Sulfamethoxazole-trimethoprim (SXT)	4	3.6	3	10.0	1	3.6	0	0
Tetracycline (TCY)	9	8.2	4	13.3	1	3.6	4	7.7

## Data Availability

The Excel spreadsheet data used to support the findings of this study are available from the corresponding author upon request.

## References

[1] Pessoa R. B. G., de Oliveira W. F., Marques D. S. C. (2019). The genus *Aeromonas*: a general approach. *Microbial Pathogenesis*.

[2] Persson S., Al-Shuweli S., Yapici S., Jensen J., Olsen K. (2015). Identification of clinical *Aeromonas* species by rpoB and gyrB sequencing and development of a multiplex PCR method for detection of *Aeromonas hydrophila*, *A. caviae*, *A. veronii*, and *A. media*. *Journal of Clinical Microbiology.*.

[3] Igbinosa I. H., Igumbor E. U., Aghdasi F., Tom M., Okoh A. I. (2012). Emerging *Aeromonas* species infections and their significance in public health. *The Scientific World Journal*.

[4] Bello-López J. M., Cabrero-Martínez O. A., Ibáñez-Cervantes G. (2019). Horizontal gene transfer and its association with antibiotic resistance in the genus *Aeromonas* spp. *Microorganisms*.

[5] Zhou Y., Yu L., Nan Z. (2019). Taxonomy, virulence genes and antimicrobial resistance of *Aeromonas* isolated from extra-intestinal and intestinal infections. *BMC Infectious Diseases*.

[6] Hiransuthikul N., Tantisiriwat W., Lertutsahakul K., Vibhagool A., Boonma P. (2005). Skin and soft-tissue infections among tsunami survivors in southern Thailand. *Clinical Infectious Diseases*.

[7] Guerra I. M. F., Fadanelli R., Figueiró M. (2007). *Aeromonas* associated diarrhoeal disease in South Brazil: prevalence, virulence factors and antimicrobial resistance. *Brazilian Journal of Microbiology*.

[8] Bhowmick U., Bhattacharjee S. (2018). Bacteriological, clinical and virulence aspects of *Aeromonas*-associated diseases in humans. *Polish Journal of Microbiology.*.

[9] Tomás J. M. (2012). The main *Aeromonas* pathogenic factors. *ISRN Microbiology*.

[10] Piotrowska M., Popowska M. (2015). Insight into the mobilome of *Aeromonas* strains. *Frontiers in Microbiology*.

[11] Zhong C., Han M., Yang P. (2019). Comprehensive analysis reveals the evolution and pathogenicity ofAeromonas, viewed from both single isolated species and microbial communities. *mSystems*.

[12] Janda J. M., Abbott S. L. (2010). The genus *Aeromonas*: taxonomy, pathogenicity, and infection. *Clinical Microbiology Reviews*.

[13] Martin-Carnahan A., Joseph S. W. (2015). *Aeromonas*. *Bergey's Manual of Systematics of Archaea and Bacteria*.

[14] Chacón M., Castro-Escarpulli G., Soler L., Guarro J., Figueras M. (2002). A DNA probe specific for *Aeromonas* colonies. *Diagnostic Microbiology and Infectious Disease.*.

[15] (2015). *Clinical and Laboratory Standards Institute, Methods for Antimicrobial Dilution and Disk Susceptibility Testing of Infrequently Isolated or Fastidious Bacteria, Third Edition Informational Supplement M45-3rd*.

[16] (2019). *Clinical and Laboratory Standards Institute, Performance Standards for Antimicrobial Susceptibility Testing; Twenty-ninth Informational Supplement M100-S29*.

[17] Heuzenroeder M., Wong C., Flower R. (1999). Distribution of two hemolytic toxin genes in clinical and environmental isolates of *Aeromonas* spp.: correlation with virulence in a suckling mouse model. *FEMS Microbiology Letters.*.

[18] Sen K., Rodgers M. (2004). Distribution of six virulence factors in *Aeromonas* species isolated from US drinking water utilities: a PCR identification. *Journal of Applied Microbiology*.

[19] Chacón M., Figueras M., Castro-Escarpulli G., Soler L., Guarro J. (2003). Distribution of virulence genes in clinical and environmental isolates of Aeromonas spp. *Antonie Van Leeuwenhoek*.

[20] Casabianca A., Orlandi C., Barbieri F. (2015). Effect of starvation on survival and virulence expression of *Aeromonas* hydrophila from different sources. *Archives of Microbiology*.

[21] da Silva L. C. A., Leal-Balbino T. C., de Melo B. S. T. (2017). Genetic diversity and virulence potential of clinical and environmental Aeromonas spp. isolates from a diarrhea outbreak. *BMC Microbiology*.

[22] Zhou H., Gai C., Ye G. (2019). *Aeromonas* hydrophila, an emerging causative agent of freshwater-farmed whiteleg shrimp Litopenaeus vannamei. *Microorganisms*.

[23] Rasmussen-Ivey C. R., Figueras M. J., McGarey D., Liles M. R. (2016). Virulence factors of *Aeromonas* hydrophila: in the wake of reclassification. *Frontiers in Microbiology*.

[24] Hoel S., Vadstein O., Jakobsen A. N. (2017). Species distribution and prevalence of putative virulence factors in mesophilic *Aeromonas* spp. isolated from fresh retail sushi. *Frontiers in Microbiology*.

[25] Citterio B., Biavasco F. (2015). *Aeromonas* hydrophila virulence. *Virulence*.

[26] Brinkmann V. (2004). Neutrophil extracellular traps kill bacteria. *Science*.

[27] Wu C.-J., Wu J.-J., Yan J.-J. (2007). Clinical significance and distribution of putative virulence markers of 116 consecutive clinical Aeromonas isolates in southern Taiwan. *Journal of Infection*.

[28] Castelo-Branco D., Guedes G., Brilhante R. (2015). Virulence and antimicrobial susceptibility of clinical and environmental strains ofAeromonasspp. from northeastern Brazil. *Canadian Journal of Microbiology*.

[29] Rather M., Willayat M., Wani S., Hussain S., Shah S. (2019). Enterotoxin gene profile and molecular epidemiology of *Aeromonas* species from fish and diverse water sources. *Journal of Applied Microbiology.*.

[30] Khor W. C., Puah S. M., Tan J. A. M. A., Puthucheary S. D., Chua K. H. (2015). Phenotypic and genetic diversity of *Aeromonas* species isolated from fresh water lakes in Malaysia. *PLOS ONE*.

[31] Iacovache I., De Carlo S., Cirauqui N., Peraro M. D., van der Goot F. G., Zuber B. (2016). Cryo-EM structure of aerolysin variants reveals a novel protein fold and the pore-formation process. *Nature Communications*.

[32] Cirauqui N., Abriata L. A., van der Goot F. G., Peraro M. D. (2017). Structural, physicochemical and dynamic features conserved within the aerolysin pore-forming toxin family. *Scientific Reports*.

[33] Oliveira S. T. L., Veneroni-Gouveia G., Costa M. M. (2012). Molecular characterization of virulence factors in *Aeromonas* hydrophila obtained from fish. *Pesquisa Veterinária Brasileira*.

[34] El-Bahar H. M., Ali N. G., Aboyadak I. M., Khalil S. A. E. S., Ibrahim M. S. (2019). Virulence genes contributing to *Aeromonas* hydrophila pathogenicity in Oreochromis niloticus. *International Microbiology*.

[35] Pereira C. S., Amorim S. D., Santos A. F. d. M. (2008). Plesiomonas shigelloides and Aeromonadaceae family pathogens isolated from marine mammals of southern and southeastern Brazilian coast. *Brazilian Journal of Microbiology*.

[36] da Saúde M. (2010). *Manual Integrado de Vigilância, Prevenção e Controle de Doen-ças Transmitidas por Alimentos*.

[37] Yano Y., Hamano K., Tsutsui I., Aue-umneoy D., Ban M., Satomi M. (2015). Occurrence, molecular characterization, and antimicrobial susceptibility of *Aeromonas* spp. in marine species of shrimps cultured at inland low salinity ponds. *Food Microbiology*.

[38] Agersø Y., Bruun M. S., Dalsgaard I., Larsen J. L. (2007). The tetracycline resistance gene tet(E) is frequently occurring and present on large horizontally transferable plasmids in *Aeromonas* spp. from fish farms. *Aquaculture*.

[39] Silva A. D. S., Barros L. S. S. E., Lima D. D. V., Velame D. S. (2019). The occurrence of bacteria of the genus *&lt;i&gt;Aeromonas&lt;/i&gt;* spp. in *&lt;i&gt;Oreochromis niloticus&lt;/i&gt;* (tilapia) and in the water of amateur sport fish ponds and sensitiveness to antimicrobials. *Food and Nutrition Sciences*.

[40] Souza G. (2015). Microbiological evaluation of water and fillets in the production chain of Nile tilapia (Oreochromis niloticus). *Journal of Aquaculture Research & Development*.

[41] Hofer E., Reis C., Theophilo G., Cavalcanti V., Lima N., Henriques M. (2006). Aeromonas associated *with* an acute diarrhea outbreak in São Bento do Una, Pernambuco. *Revista da Sociedade Brasileira de Medicina Tropical*.

[42] Chen P.-L., Ko W.-C., Wu C.-J. (2012). Complexity of *β*-lactamases among clinical *Aeromonas* isolates and its clinical implications. *Journal of Microbiology, Immunology and Infection*.

[43] Sinha S. (2004). Prevalence, serotype distribution, antibiotic susceptibility and genetic profiles of mesophilic *Aeromonas* species isolated from hospitalized diarrhoeal cases in Kolkata, India. *Journal of Medical Microbiology*.

[44] Chenia H. (2016). Prevalence and characterization of plasmid-mediated quinolone resistance genes in *Aeromonas* spp. isolated from south African freshwater fish. *International Journal of Food Microbiology.*.

[45] Varela A. R., Nunes O. C., Manaia C. M. (2016). Quinolone resistant *Aeromonas* spp. as carriers and potential tracers of acquired antibiotic resistance in hospital and municipal wastewater. *Science of The Total Environment*.

[46] Wimalasena S., De Silva B., Hossain S., Pathirana H., Heo G. (2017). Prevalence and characterisation of quinolone resistance genes in *Aeromonas* spp. isolated from pet turtles in South Korea. *Journal of Global Antimicrobial Resistance.*.

[47] Stratev D., Odeyemi O. (2016). Antimicrobial resistance of *Aeromonas* hydrophila isolated from different food sources: a mini-review. *Journal of Infection and Public Health.*.

